# Forearm sEMG data from young healthy humans during the execution of hand movements

**DOI:** 10.1038/s41597-023-02223-x

**Published:** 2023-05-20

**Authors:** Manuela Gomez-Correa, Mariana Ballesteros, Ivan Salgado, David Cruz-Ortiz

**Affiliations:** 1 Centro de Innovación y Desarrollo Tecnológico en Cómputo, Instituto Politécnico Nacional, Z.C, 07700 Mexico City, Mexico; 2 Medical Robotics and Biosignals Laboratory, Unidad Profesional Interdisciplinaria de Biotecnología, Instituto Politécnico Nacional, Z.C, 07340 Mexico City, Mexico

**Keywords:** Biomedical engineering, Occupational health

## Abstract

This work provides a complete dataset containing surface electromyography (sEMG) signals acquired from the forearm with a sampling frequency of 1000 Hz. The dataset is named WyoFlex sEMG Hand Gesture and recorded the data of 28 participants between 18 and 37 years old without neuromuscular diseases or cardiovascular problems. The test protocol consisted of sEMG signals acquisition corresponding to ten wrist and grasping movements (extension, flexion, ulnar deviation, radial deviation, hook grip, power grip, spherical grip, precision grip, lateral grip, and pinch grip), considering three repetitions for each gesture. Also, the dataset contains general information such as anthropometric measures of the upper limb, gender, age, laterally of the person, and physical condition. Likewise, the implemented acquisition system consists of a portable armband with four sEMG channels distributed equidistantly for each forearm. The database could be used for the recognition of hand gestures, evaluation of the evolution of patients in rehabilitation processes, control of upper limb orthoses or prostheses, and biomechanical analysis of the forearm.

## Background & Summary

Electromyography (EMG) signals are the electrical activity of the muscles during spontaneous or voluntary contraction processes. Also, these signals give information about nerve action potentials caused by the inducement of peripheral nerves^1^. Amplitude and frequency are the most relevant aspects in the study of EMG signals. For amplitude, the EMG contemplates the range of 50 *μ*V to 100 *μ*V; this parameter allows us to identify the degree of muscle activation and the time this action takes^2,3^. In the case of frequency, the main components are between 10 to 500 Hz^4^. The frequency permits the evaluation of fatigue levels^2^. EMG signals can be measured by invasive methods, which use needle electrodes, and non-invasive techniques, considering surface electrodes placed on the skin^5^; this last case is known as surface electromyography (sEMG).

Particularly, the measurement of EMG signals has been implemented for disease diagnosis^6^, development of prosthetic devices^7^, and biomechanical analysis^5,8^. Likewise, recent studies have shown that various parameters extracted from these signals help to establish a quantitative index of rehabilitation progress since they allow evaluating the effectiveness and quality of the functioning of physiological processes related to the mobility of the person^9^. Specifically, sEMG of the forearm has been used for different applications, such as assisted mobility using an instrumented glove, the development of actuators for soft hand exoskeletons, and remote control of robotic arms^10^.

Multiple databases have recorded sEMG signals of the forearm from complex and non-portable acquisition systems. One example of such a database is the one presented by Xinyu Jiang *et al*.^11^, in which high-density sEMG signals were acquired from the forearm of 20 subjects through a 256 channel sensors during dexterous finger manipulations, using an elaborated and expensive acquisition system, which limits the reproducibility of the data acquisition and therefore its application in further research. Another example is given in the work of Nesto J. Jarque-Bou *et al*.^12^, where the authors generated a calibrated database of the kinetics and sEMG signals of the forearm and hand during activities of daily living. For the acquisition of these signals, an instrumented glove was used for the registration of 18 hand anatomical angles, and sEMG sensors were implemented for the acquisition of the signals locating the electrodes in seven regions of interest of the arm, with a sampling frequency of 1000 Hz, and bandwidth from 20 Hz to 406 Hz. However, for the location of the electrodes, it was necessary to identify 30 specific points on the person’s forearm. Therefore, since the glove was made up of gauges to be able to measure angles on the hand, there could be inaccuracies because this has a standard measurement that does not adjust itself to the anatomy of each person.

Additionally, Ashirbad Pradhan *et al*., recorded an EMG dataset from 43 participants^13^. In the acquisition process were placed 28 monopolar sEMG electrodes in the form of four rings around the forearm, which caused high-time consumption in the acquisition protocol and limited large-scale data collection. Mariusz P. Furmanek *et al*., generated a kinematic and sEMG dataset of online adjustment of reach-to-grasp movements to instantaneous perturbations with 20 participants using a virtual environment for kinematic measurement and EMG sensors applying a specific and thorough protocol^14^. These aspects caused the low reproducibility of the data acquisition. Based on the aforementioned, we formulate the following research question for our work:

**Which factors can be considered to produce a dataset with information on basic hand movements and dexterity from young adults between 18 and 37 years old that can be easily reproducible and contains sufficient information for applications such as classification or identification?**

Answering our research question, we develop this work in which the acquisition of sEMG signals of the forearm is presented using a low-cost system and an acquisition protocol with high reproducibility, with the final purpose of acquiring and using sEMG signals for its implementation in the design and validation of new applications. For this, the WyoFlex armband was used, which has four channels and a sampling frequency of 1000 Hz^15^. Likewise, the data contemplate 28 subjects without any pathology in the upper extremities recorded. The data included the execution of ten grasping movements of both forearms, collecting three repetitions for each participant.

**The main contributions of this dataset are:**

- The data is from an open-source and low-cost device which might allow the replicability in further acquisition processes.
- The number of subjects chosen in this study was based on the analysis of different current datasets. In forearm sEMG databases such as those acquired in^16–19^, there are a low number of test subjects, which may condition some analyses. Thus, having information on 28 subjects, from which up to six trials were obtained (three for each forearm), there is sufficient information for the development of different analyzes in applications such as rehabilitation, classification, identification algorithms, prosthesis, or orthosis control, among others.
- On the other hand, several databases focus on acquiring signals of basic movements, such as flexion, extension, and fisting, without considering the purpose for which the movements could be used. For this reason, the signals acquired in the present work contemplate the four basic hand gestures and the movements classified by Schlesinger as the types of basic grips of the functional hand^20^.
- Finally, most databases carry out the signals acquisition at strategic points, with a great number of sensors or using very specialized equipment, making it harder to work with this type of signal since they are not acquired under the same conditions. This limitation is reduced considering that the database was generated with an equidistant arrangement of the sensors, contemplating that the armband is placed in the middle of the forearm, and the database has information on the conditions in which the signals were acquired, facilitating the implementation of these in multiple studies.

## Methods

### Participants

The participants were selected according to the inclusion protocol SIP-20221503 approved by the research committee and regulated by the research and postgraduate secretariat (Secretaría de Investigación y Posgrado del Instituto Politécnico Nacional). The inclusion criteria set healthy participants without any neuromusculoskeletal, cardiovascular, pulmonary, or neurological diseases. In addition, the participants can be subjects of both sexes, males or females, between 18 and 37 years old, from any race, religion, and ethnic orientation. For this study, the data from 28 participants within the ages stipulated in the protocol was considered to create the database, Table 1 shows a summary of the participants in this study.

**Table 1:** General information of the participants in the database.

Participant	Age	Physical activity	Gender	Laterality
1	22	1–2 days per week	Male	Right
2	19	Sedentary	Female	Right
3	31	Sedentary	Male	Right
4	31	Sedentary	Male	Right
5	21	3–5 days per week	Male	Right
6	21	1–2 days per week	Male	Right
7	21	3–5 days per week	Male	Right
8	33	3–5 days per week	Male	Right
9	37	1–2 days per week	Male	Right
10	24	Sedentary	Male	Right
11	32	Sedentary	Male	Right
12	21	1–2 days per week	Male	Right
13	22	Sedentary	Female	Left
14	36	Sedentary	Female	Right
15	22	1–2 days per week	Male	Right
16	26	3–5 days per week	Male	Right
17	20	3–5 days per week	Male	Right
18	20	3–5 days per week	Female	Right
19	24	1–2 days per week	Female	Right
20	19	1–2 days per week	Male	Right
21	19	Sedentary	Male	Right
22	35	3–5 days per week	Male	Right
23	19	3–5 days per week	Male	Right
24	20	Sedentary	Female	Right
25	21	1–2 days per week	Female	Right
26	22	Sedentary	Female	Ambidextrous
27	22	Sedentary	Male	Right
28	30	3–5 days per week	Female	Right

### Instrumentation

**Fig. 1:**
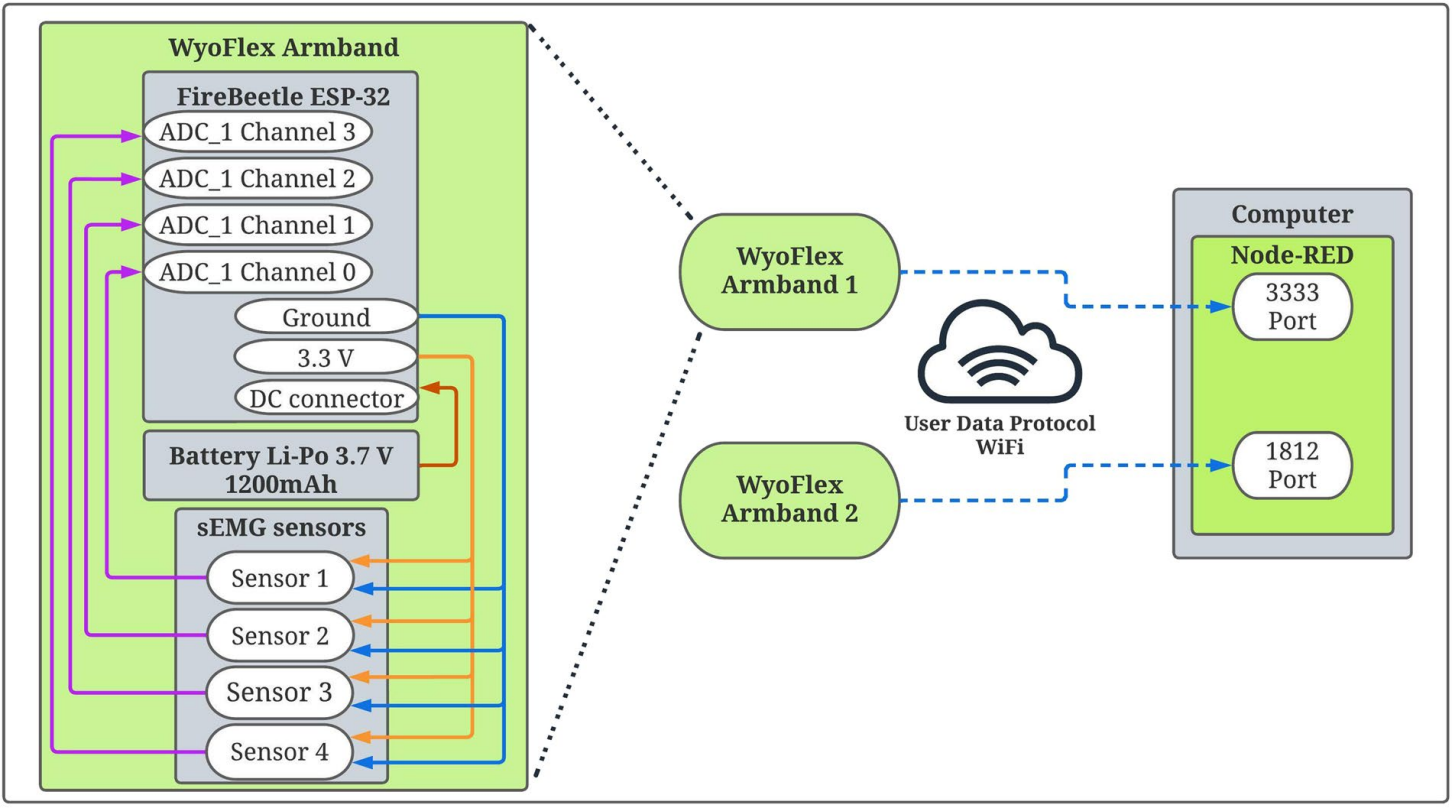
Instrumentation applied to perform the sEMG data measurement. It comprises the sEMG sensors, the microcontroller board to process the information and the application programming interface (API) developed with the Node-Red tool.

The acquisition stage implements the WyoFlex armband^15^. This device is a wearable sEMG system for remote biosignals acquisitions. Figure 1 describes the complete instrumentation to obtain the sEMG signals from the participants. In this case, the considered electronic instrumentation has three main stages, the acquisition stage with the sEMG sensors; the signal processing and amplifying stage; and the transmission stage. In the first stage, each sEMG acquisition system (WyoFlex armband) has four Gravity Analog sEMG sensors. Each sensor has two modules; a dry electrode board comprises the first module. The electrodes have a differential input, high common mode rejection ratio, low power consumption, and single power supply. The second module has electronic elements for amplifying and transmitting the obtained signals. Table 2 summarizes the characteristics of the transmitter board. A total of eight sensors were employed to measure the data from the volunteers, four for each armband and one armband for each forearm.

**Table 2:** Signal transmitter board features.

Feature	Value
Supply Voltage	3.3 V ~5.5 V
Operating Voltage	3.3 V
Detection Range	±1.5 mV
Output Voltage	0~30 V
Operating Temperature	0~50 °C

Based on previous works regarding sEMG acquisition^21–25^, in the second stage, to perform the measurements, the dry electrode board was placed around the circumference of the forearm. The sampled rate of each sEMG sensor was selected as 1000 Hz (considering that the dominant range of the sEMG is from 10 to 500 Hz^4^), with a 12-bit analog-digital converter (ADC) implemented in the FireBeetle ESP32 microcontroller. The microcontroller supports two power supply methods: USB and 3.7 V external lithium battery. Then, four ADC channels converted the analog information to its digital counterpart to be transmitted using an User Datagram Protocol (UDP) to a personal computer, where a graphical user interface (GUI) based on the Node-RED tool performed the data visualization and storage^26^. For more details about the electronic instrumentation of the WyoFlex device the readers are referred to the work developed by Manuela Gomez-Correa and David Cruz-Ortiz^15^.

### Experimental protocol

In order to carry out the signal acquisition, this work considered the following four relevant aspects to design the experimental protocol, which defines how the signals should be acquired.

1. Selected movements
2. Acquired metadata
3. Number of participants
4. Homogeneity of the signals

First of all, it was determined the four basic hand movements: flexion, extension, ulnar deviation, and radial deviation. These gestures are executed mainly by the superficial muscles of the forearm, which are the main contributors to the signals acquired by the sEMG sensors that make up the WyoFlex armband, as shown in Fig. 1. Furthermore, six additional movements were selected according to the types of grip defined by Schlesinger in the study of hand dexterity for the upper limb taxonomy^20^. Therefore, the chosen six main grasps were the power grip, precision grip, hook grip, lateral grip, spherical grip, and pinch grip.

Once the movements were defined, it was determined to perform not only the recording of sEMG signals in the subjects but also the acquisition of information such as gender, age, physical activity of the people, and anthropometric measurements of the upper limb. With this information, it is possible to carry out a classification analysis, for example, evaluating the possible implications of the armband placement distance, such as differences in the signals according to gender and the implications of the physical activity conditions in the relevant characteristics of this type of signals such as the amplitude or the frequency.

Likewise, different databases were evaluated for the choice of the number of participants. According to the literature, in most published datasets, the acquisition of sEMG signals contemplates from 5 to 18 users. Because of this, and in order to create a broader database for different analyses, it was determined that taking 28 participants was an appropriate number for the established acquisition protocol considering that each executes six trials and three cycles for each forearm.

The last aspect considered was the homogeneity of the signals. Then, a tutorial was designed to show step-by-step how the test should be executed. The tutorial shows a participant performing the same test in such a way that the user can follow it simultaneously. With the above, it was possible to generate a homogeneous acquisition, considering that this method allows better control of the speed and execution time of each hand gesture. Also, studies like the one developed by María V. Arteaga were contemplated for the protocol^18^. Their protocol considered six seconds for the execution of each movement, three seconds to make the gesture, and three seconds for rest. Additionally, to acquire multiple trials of the same test subject, it was obtained that the signals for both forearms were simultaneous, performing three cycles per person. With this information, it could be possible to perform laterality and fatigue analysis.

**Fig. 2:**
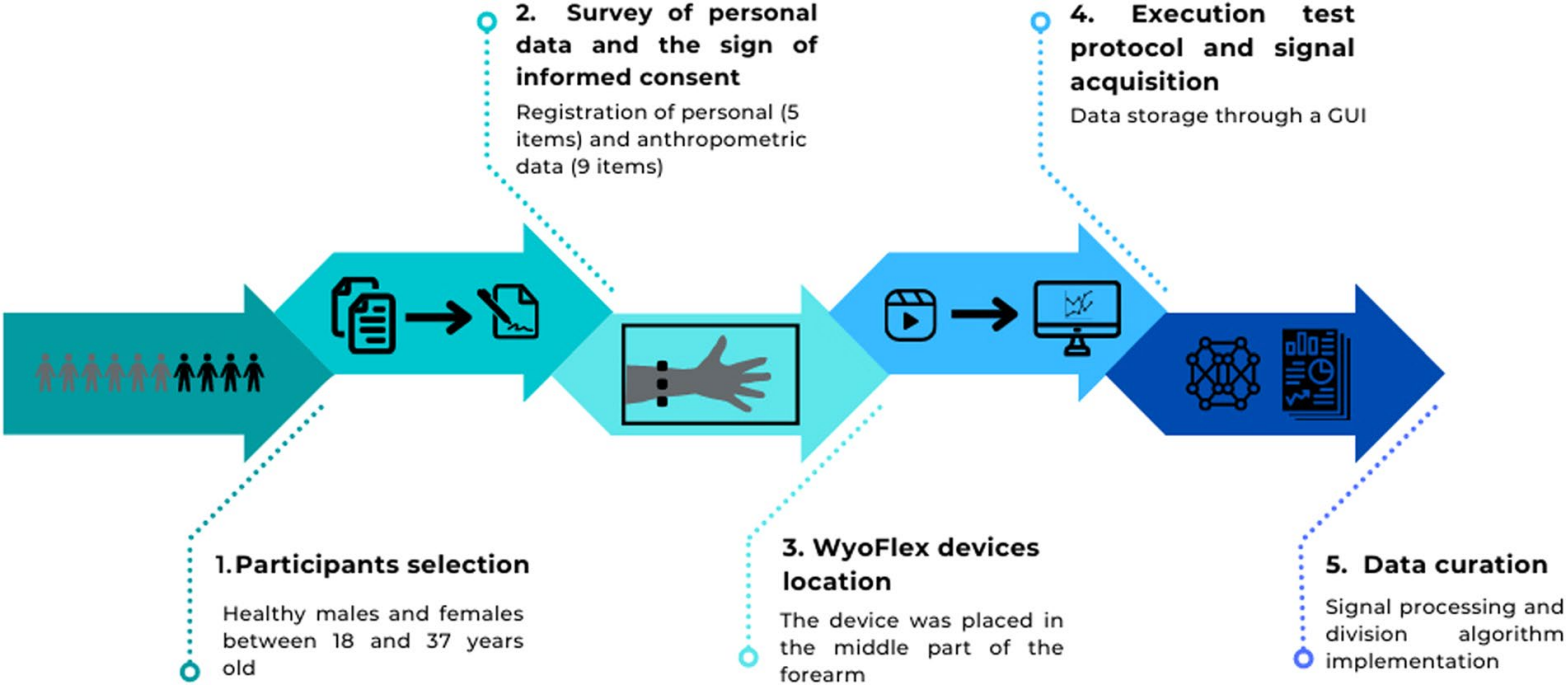
General scheme of the experimental protocol stages.

Based on the previous facts, the proposed experimental protocol is divided into five main stages. The first one is the participant selection. Then, the second stage is the survey of personal data and the sign of informed consent. The third step considers the location of the WyoFlex device. The execution test protocol by the participant and signal acquisition integrate the stage four. Finally, the last stage describes the data curation. To sum up all the steps in the experimental protocol, Fig. 2 provides an overview of the proposed experimental protocol to obtain the dataset. A detailed description of each section is provided below.

#### Stage 1

The first stage of the experimental protocol is the participant selection. Here, it is corroborated that each female or male who wants to participate in the test is in the 18–37 age range. Also, it is verified that the participant is a healthy person without any neuromusculoskeletal problem or cardiovascular, pulmonary, or neurological disease.

#### Stage 2

In order to collect personal data such as name, age, as well as anatomical measurements of the upper limb, we designed a survey. The survey was filled out after the informed consent was obtained from each participant. Table 3 presents the information acquired through the survey with the nomenclature used for each parameter. Then, at the beginning of the test protocol, anthropometric information was measured with a measuring tape. Some of the obtained results are summarized in Table 4. Then, two WyoFlex armbands (one per each forearm) were used for data recording of the sEMG signals.

**Table 3:** Personal information.

Information	Acquired data	Nomenclature
General information	Age	Age
	Physical activity	Physical activity
	Gender	Gender
	Laterality	Laterality
Anthropometric dimensions	Arm length	AL
	Forearm length	FL
	Maximum arm circumference	MAC
	Minimum arm circumference	mAC
	Maximum forearm circumference	MFC
	Minimum forearm circumference	mFC
	Mid arm circumference	iAC
	Mid forearm circumference	iFC
	Distance between the armband location and the elbow	AE

**Table 4:** Anthropometric dimensions. All units in centimeters.

Participant	AL	FL	MAC	mAC	MFC	mFC	iAC	iFC	AE
1	32.0	28.0	37.0	28.5	28.0	16.0	31.5	23.5	13.0
2	29.5	26.0	30.0	24.5	24.0	17.0	26.0	21.5	7.5
3	30.0	27.0	40.0	29.0	28.0	18.0	33.0	24.0	14.5
4	34.0	26.0	41.0	31.0	29.0	17.0	34.0	25.0	14.0
5	28.0	25.0	35.0	29.0	26.5	17.0	30.0	25.0	14.0
6	32.0	26.0	30.0	23.5	24.5	15.0	25.0	20.5	9.5
7	33.0	27.5	30.5	26.0	26.0	16.5	28.5	23.0	12.0
8	27.0	23.0	32.0	28.0	26.5	17.0	31.5	24.0	11.0
9	28.0	26.0	33.5	29.0	29.0	19.0	35.0	24.0	15.0
10	29.0	28.0	34.0	27.0	27.0	16.0	29.5	25.5	13.0
11	29.5	23.0	35.0	27.5	25.0	16.0	30.0	20.0	10.0
12	33.0	26.0	32.0	29.0	28.5	18.0	31.0	25.5	13.0
13	29.0	26.0	29.0	23.5	22.5	14.0	24.5	18.5	9.0
14	29.5	25.5	35.0	25.0	24.5	15.0	27.5	21.5	10.0
15	32.0	28.0	34.0	29.0	27.0	17.0	30.5	23.5	14.0
16	34.0	26.5	34.0	23.0	23.0	15.0	27.0	21.0	8.0
17	28.5	25.5	31.0	24.0	25.5	16.5	27.5	22.0	11.5
18	33.0	28.0	29.0	24.0	24.0	16.0	25.0	22.0	10.5
19	28.0	23.0	34.0	25.0	23.5	15.0	28.5	21.0	9.5
20	31.0	25.0	31.5	27.0	26.0	17.0	29.0	26.0	13.0
21	29.0	27.0	30.0	23.0	24.0	16.0	25.5	21.0	8.0
22	33.0	25.0	37.0	29.0	28.0	17.0	32.0	24.5	13.5
23	33.0	24.5	30.5	25.0	26.0	16.0	28.5	22.5	12.0
24	29.0	27.0	30.0	23.0	23.5	15.0	27.0	19.5	11.0
25	29.5	23.0	35.0	27.5	25.0	16.0	30.0	20.0	10.0
26	27.0	22.5	31.0	25.0	24.0	15.5	28.5	22.0	8.0
27	32.0	25.0	34.0	25.5	26.0	16.0	29.0	19.5	12.5
28	25.0	23.0	29.5	22.0	22.0	14.0	24.0	20.0	12.5

#### Stage 3

In order to place the sEMG sensors, this study considered the recommendations provided in the guide entitled European recommendations for sEMG (SENIAM)^27^. Therefore, each WyoFlex device was placed in the middle of the forearm based on the recommendations obtained from a literature review^21–25^. However, it should be emphasized that even when the middle of the forearm was the suggested location, if the forearm circumference of the participant was smaller than the diameter of the WyoFlex armband, the device had to be displaced to an upper part of the forearm in order to guarantee the correct contact between the electrodes and the participant skin.

**Fig. 3:**
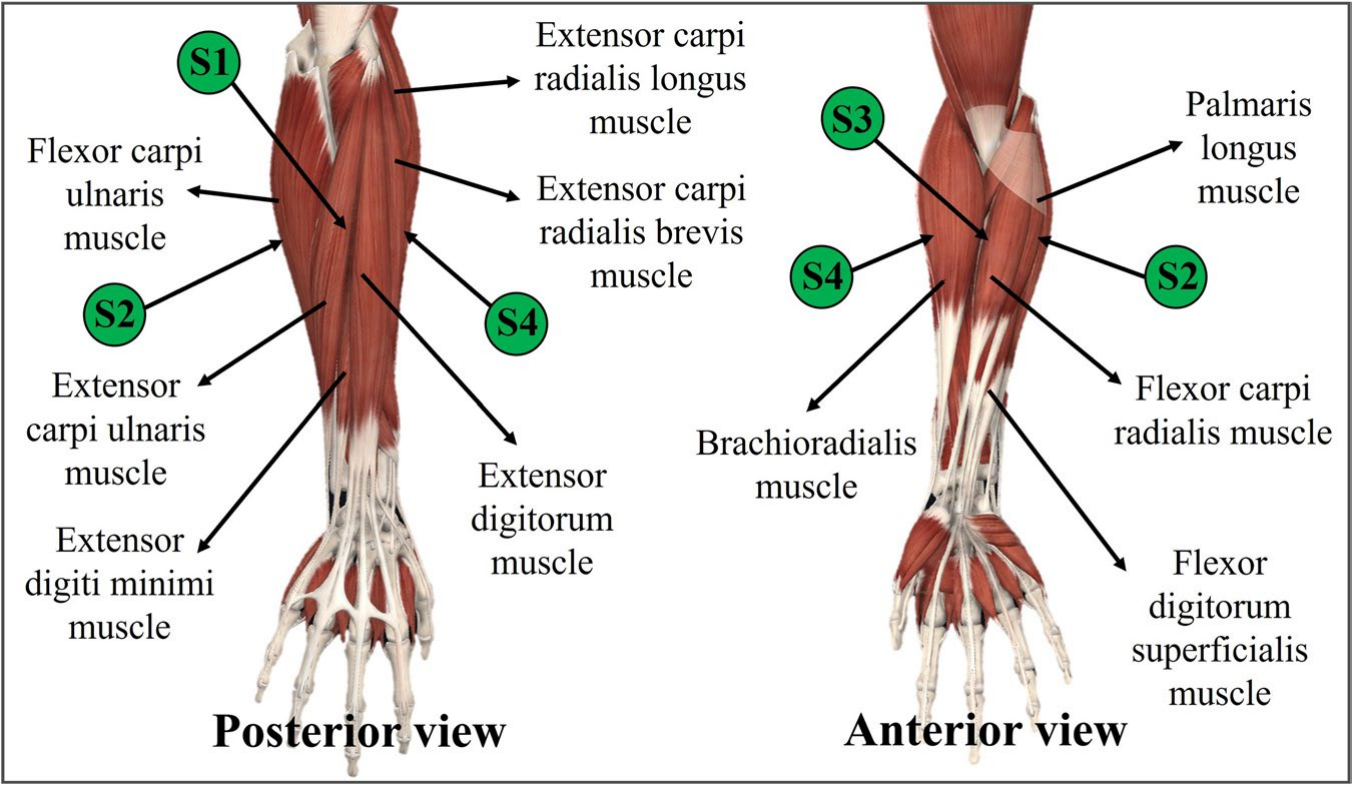
Location of the sEMG sensors in the forearm.

Four sEMG sensors were placed on the intended muscles of each forearm. In this case, Sensor 1, labeled as S1 was located over the posterior part of the forearm, which corresponds to the Exterior digitorium muscle and Extensor carpi ulnaris muscle. Correspondingly, Sensor 2 (S2) was placed over the external side of the forearm, that is, over the muscles Palmaris longus and Flexor carpi ulnaris. Then, Sensor 3 (S3) was placed over the Brachioradialis muscle and Flexor carpi radialis muscles. Finally, the last Sensor (S4) was located in the position corresponding to the Extensor carpi radialis longus and the Extensor carpi radialis brevis muscles. To sum up the sensor location of the WyoFlex device, Fig. 3 evidences the location of each sensor.

#### Stage 4

**Fig. 4:**
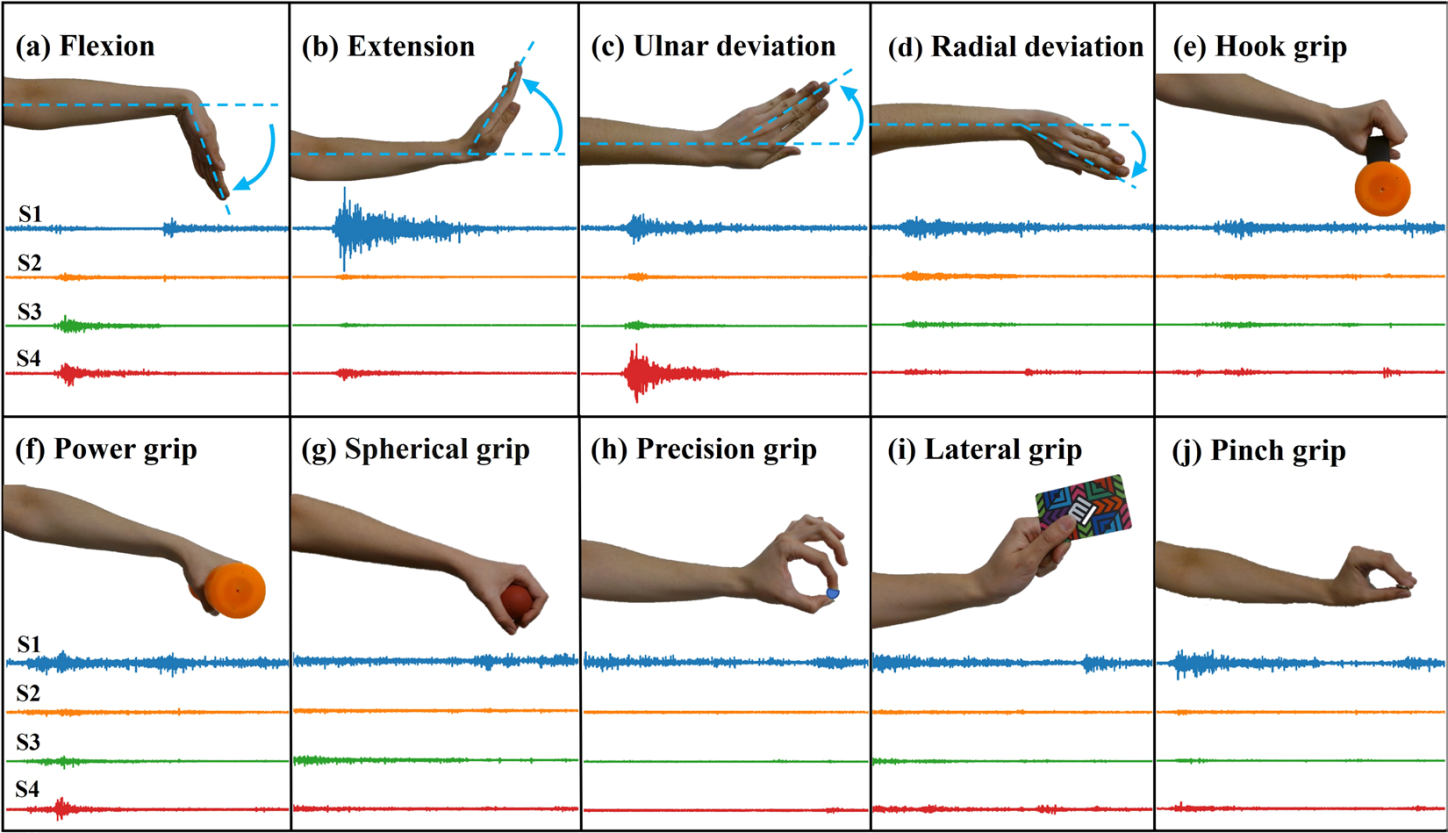
Movements executed in the test protocol with its corresponding sEMG signals.

Once the four electrodes were placed in the correct position, it was verified that each electrode had enough contact with the participant’s skin. For that, it was corroborated that the baseline of the sensors was 1.5 V through the GUI. Then, all the participants were instructed to perform ten different hand movements: flexion, extension, ulnar deviation, radial deviation, hook grip, power grip, spherical grip, precision grip, lateral grip, and pinch grip to obtain its corresponding sEMG of each of the four sensors (S1, S2, S3, and S4). As an example, Fig. 4 shows the ten mentioned movements aside from an example of the corresponding sEMG obtained signals. Then, aiming that the participants execute the correct form of the required movements, a video tutorial showing the movements that the participants should perform during the experimental protocol was created.

**Fig. 5:**
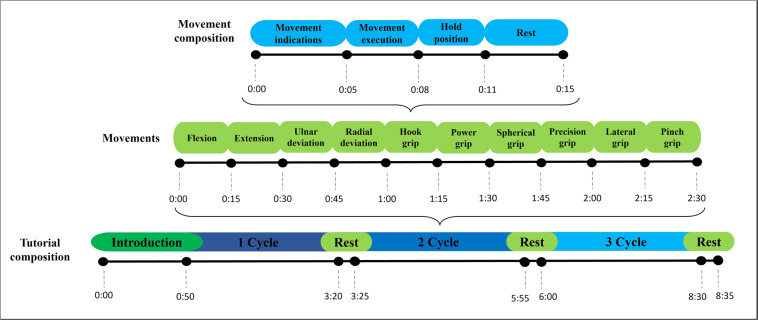
Video tutorial structure.

The video tutorial lasts eight minutes and 35 seconds distributed equally on three cycles (see the video tutorial structure in Fig. 5). In this case, each test allows the acquisition of 24 sEMG signals per participant (four sEMG signals for each band during three cycles). With the participant seated, preserving 90 degrees between all lower limb joints, the tutorial starts with an introduction section, where a general explanation of the test is provided to the participant (first 50 seconds of the tutorial). Then, the participant starts with the first cycle, which consumes around 200 seconds. Here, it should be emphasized that the participant has 15 seconds to execute each of the ten movements (see the movements section in Fig. 5).

The 15 seconds are distributed as in the movement composition section of Fig. 5. In the first five seconds, the movement that the patient should execute is shown in the video tutorial (movement indications in Fig. 5). Then, the following three seconds are considered to execute the movement. After that, the participant must maintain the position for three seconds. Finally, the participant has four seconds to rest and continue to the next action. Notice that, at the end of each cycle, the participant has five extra seconds to rest after continuing with the second and third cycles. At this point, all the information data vectors generated during the execution of the test protocol are sent to the GUI.

Notice that the participants should execute the test comfortably and look ahead to the monitor where the tutorial was played. If the participants report fatigue during the test or execute the movements in an incorrect form, the trial should be rejected.

#### Stage 5

The data of each signal is sent through a message (from the microcontroller to the GUI) containing a character *A* to identify the start of the vector information and four characters representing the ADC value of each sEMG sensor. Thus, each data vector is integrated with 17 characters (one for character A, and four for each sensor). Equation (1) describes the vector information structure.

1 $${V}_{I,ki}=\left\{A,{S}_{1,ki},{S}_{2,ki},{S}_{3,ki},{S}_{4,ki}\right\},$$

where *S*_*j, ki*_ represents ADC data from the *j*-th sensor with *j*={1, 2, 3, 4}, the variable *k* = {*I, D*}, refers to the WyoFlex located in the left and right forearm, respectively; and the subscript *i* = {1, 2, 3,..., *n*} denotes the *i*-th number of sample in the recorded sEMG signals. All the data vectors are received in the GUI designed in the Node-RED environment. The acquired signals consider an offset in their amplitude (a digital value of 1862, according to the sensor manufacturer). This means that the signals can vary in a digital value range from 0 to 4095 due to the ADC module resolution. Notice that even when the sensor manufacturer recommends considering a digital value of offset 1862 (or approximate 1.5 V), the authors corroborate in experimental tests that an offset value of 1756 should be selected to generate sEMG signals with a baseline on zero. In this particular case, the proposed offset corresponds to a mean value of all the acquired data vectors.

### Dataset elaboration

**Fig. 6:**
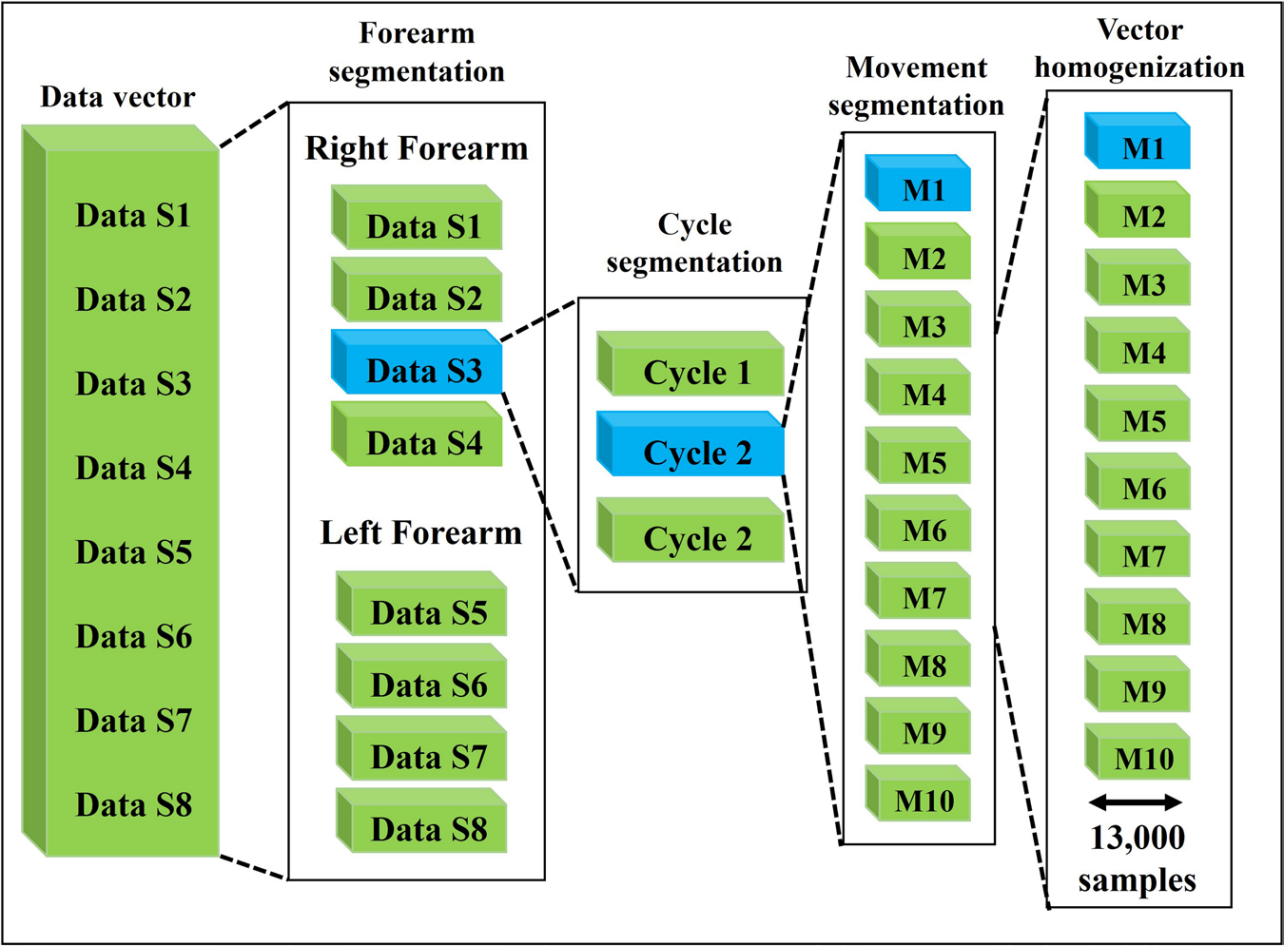
The scheme shows a graphical representation of the steps in the data segmentation algorithm. In the scheme’s first column, the data vector is provided as input for the segmentation algorithm. Then, the four mentioned stages are forearm segmentation, cycle segmentation, movement segmentation, and vector homogenization.

As the final step on the GUI, the user obtains a data vector, which contains the sEMG signals of each of the eight sensors as can be observed in the first part of the scheme given in Fig. 6. This data vector is stored by the GUI in a comma-separated values (CSV) file. Then, in order to obtain the dataset, a data segmentation algorithm is implemented to separate the data of each sensor and store it in vectors for the sEMG signals offline visualization.

#### Data segmentation algorithm

A segmentation algorithm is implemented in Python to obtain homogeneous data vectors of each sEMG signal corresponding to each hand movement. Here, the algorithm considers four stages: forearm segmentation, cycle segmentation, movement segmentation, and vector homogenization. Figure 6 shows a block diagram where each column corresponds to the four mentioned stages in the segmentation algorithm. The blue color in some blocks of Fig. 6 denotes some examples (shown below) of the signals obtained in that particular stage of the segmentation algorithm.

#### Stage 1: Forearm segmentation

**Fig. 7:**
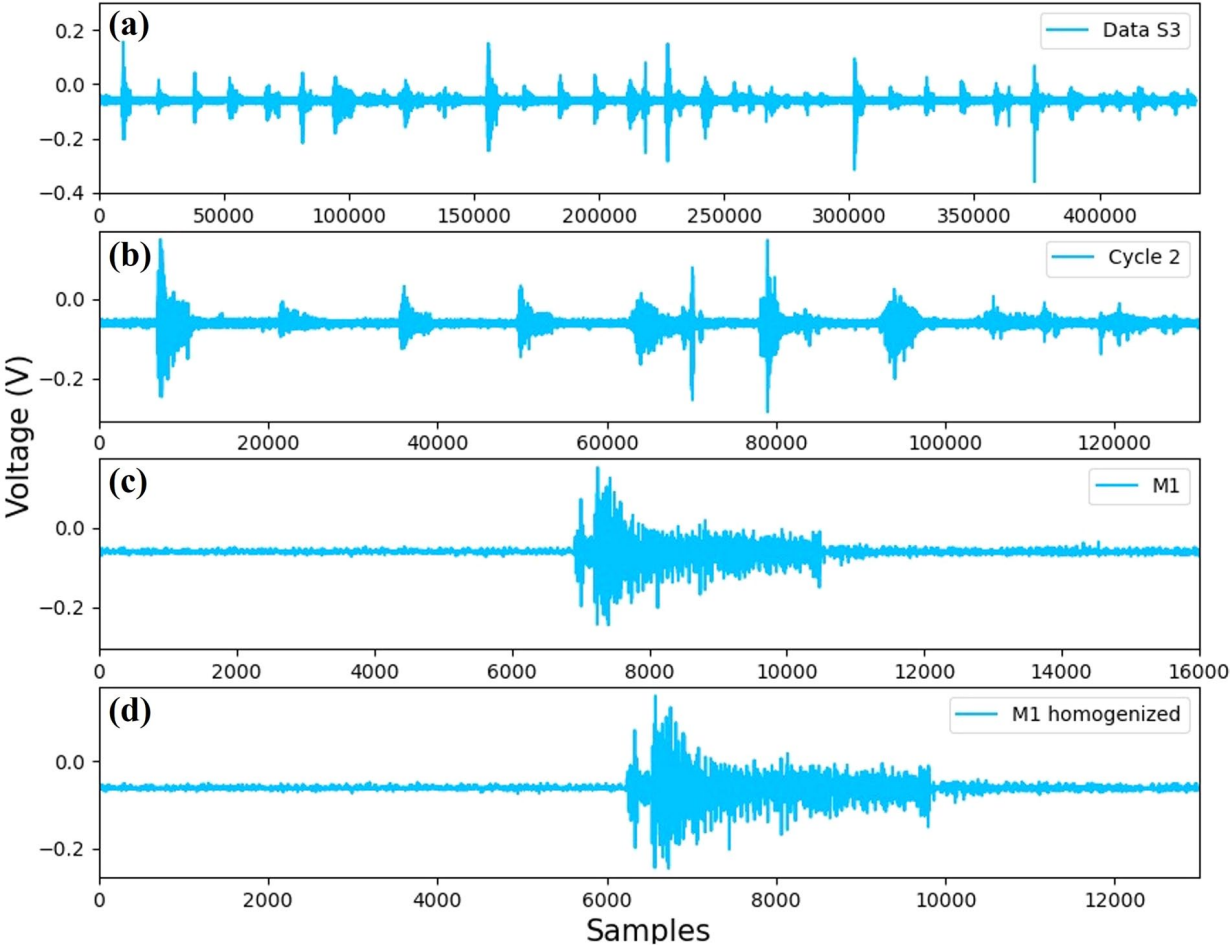
Example of the obtained signals in each of the first three stages of the segmentation algorithm considering the Data S3.

The algorithm segmentation starts when the CSV file containing the eight sensors information separated by semicolons is loaded. Then, each data vector is divided into eight sub-vectors, that is, four for the left forearm and four for the right forearm (see Fig. 7a).

#### Stage 2: Cycle segmentation

The subsequent step is in charge of dividing the data vector into three cycles per sensor (see Fig. 7b). To this end, the segmentation algorithm considers the data information measured from the sensor *S*_1_, which in this particular case is established as a reference for the segmentation. This sensor is selected due to the characteristic (maximum) amplitudes generated during the execution of the extension movement. Notice that, to divide the cycle it is also considered the time provided by the video tutorial in order to improve the information synchronization.

#### Stage 3: Movement segmentation

This step consists of obtaining the ten movements per cycle, that is, one vector for each movement (see Fig. 7c).

#### Stage 4: Vector homogenization

After the movement segmentation stage, the vector homogenization is executed. The main objective of this step is to generate six vectors containing 13000 data points (see Fig. 7d). To this end, the algorithm calculates the difference between the length of the motion vector and 13000 samples. Then, half of the difference at the beginning of the vector is deleted, and the other half of the difference is deleted at the end.

At this point of the data segmentation algorithm, the corresponding sEMG signal of each movement is obtained. To improve the readability of the effect of each of the described stages, Fig. 7 has been added. This figure shows the signals obtained after implementing the four stages of the segmentation algorithm following the sequence of the blue blocks in Fig. 6.

Once the segmentation algorithm is finished, the sEMG of each motion is stored also in a CSV file with specific labels according to the movement to which they correspond. Here, it should be emphasize that two types of CSV files are obtained as output of the algorithm segmentation. The first files contain the sEMG signals in digital value, whereas the second ones contain the signals in voltage value. Notice that, in both cases, the dataset considers signals with and without amplitude offset. In the section given below a detailed explanation about the information in the dataset is provided.

## Data Records

The WyoFlex sEMG Hand Gesture dataset is available for download at *figshare*^28^. The signals are CSV files storaged into two folders **DIGITAL DATA** and **VOLTAGE DATA** containing the signals obtained after the segmentation algorithm. Here, as the name of the folders suggests, the difference between both folders is that **DIGITAL DATA** contains signals in ADC value. In contrast, **VOLTAGE DATA** contains the sEMG signals in volts. In both folders, the user can find two types of signals, with and without offset, which means that the baseline of the signals is zero (with offset) or different from zero (without offset).

In order to simplify the loading of the dataset, the files are named following the subsequent nomenclature *P*#*C*#*S*#*M*#*B*#*F*#, where each character # should be replaced by numbers according with the following rules.

- *P* describes the subject number. Since, this study considers 28 participants, *P* can take values from 1 to 28
- *C* is the cycle number, *C* can take the values from 1 to 3
- *S* is the sensor number, *S* can take the values from 1 to 4
- *M* denotes the executed movement, *M* can take the values from 1 to 10. That is, 1 to flexion, 2 to extension, 3 to ulnar deviation, 4 to radial deviation, 5 to hook grip, 6 to power grip, 7 to spherical grip, 8 to precision grip, 9 to lateral grip, and 10 to pinch grip
- *F* denotes the left or right forearm, *F* can take the value 1 for right or 2 for left
- *O* denotes if the signal has offset or not. Then, *O* can be 1 for a signal with offset or 2 for a signal without offset

**Fig. 8:**
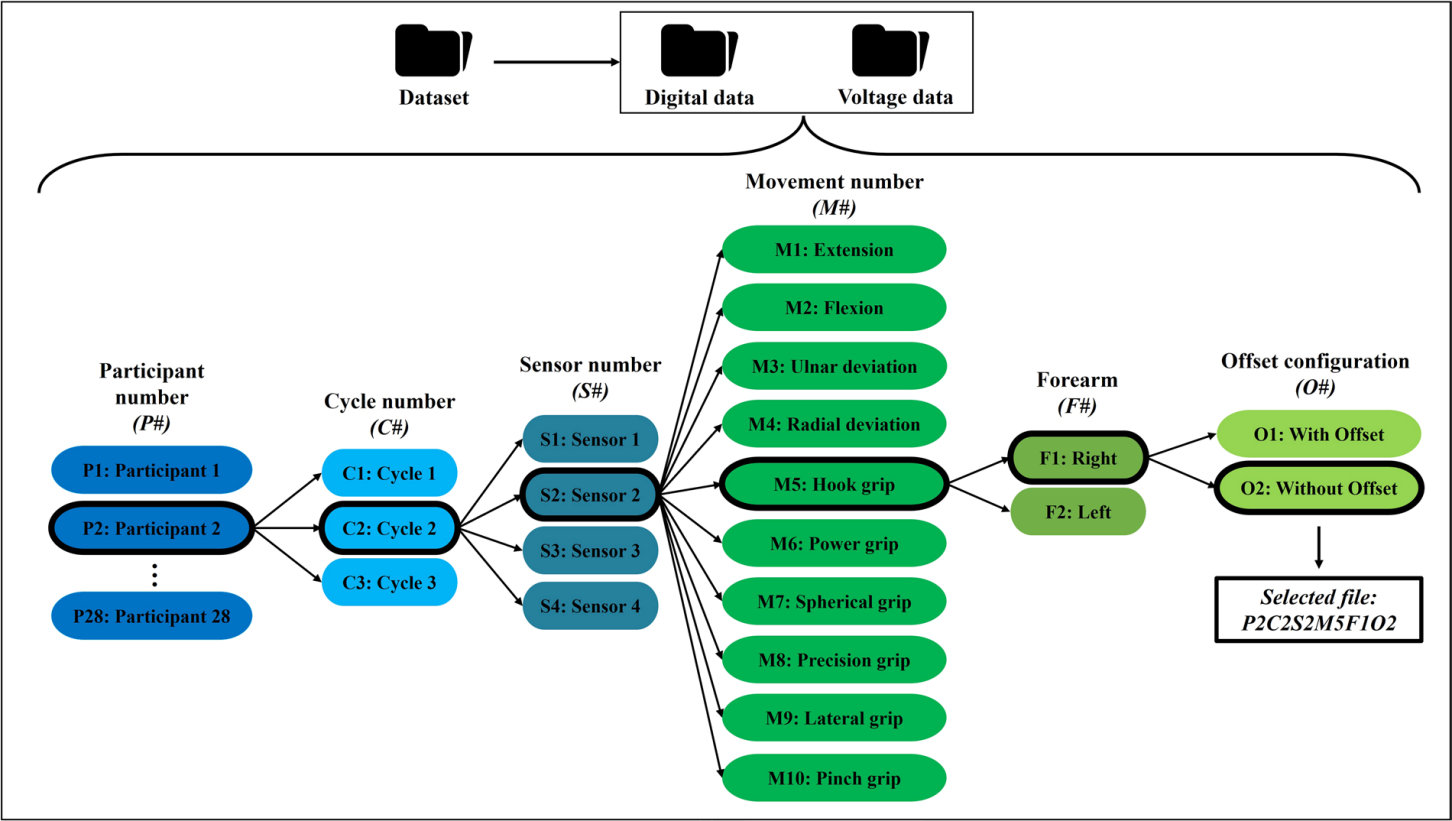
Dataset organization in the proposed database.

To sum up the dataset organization, Fig. 8 shows a general diagram explaining how the data set files are organized. In this figure, the blocks with the bold line indicate the selection of the file *P*2*C*2*S*2*M*5*F*1*O*1, which corresponds to the signal obtained from participant two, in cycle two, with sensor two, executing the hook grip movement with the right forearm. The corresponding signal has an offset, meaning the baseline equals zero.

### Metadata

The *Metadata.xls* file contains the following information: (i) the participant’s identification defined as *ParticipantX*, where *X* varies from 1 to 28; (ii) *Age*, the participant’s age in years; (iii) *Physical activity*; (iv) *Gender*, the participant’s gender (Male or Female); (v) *Laterality*; (vi) *Anthropomorphic dimensions* in meters. Table 4 shows a summary of the anthropomorphic dimensions of all the participants in this study. The aim of including each item in the metadata is to provide the dataset users with as much information as possible about the participants. For example, regarding age, the intention is to prove that the signals were acquired from young people between 18 and 37 years old. Physical activity can be used in some studies to analyze the exercise routine’s relevance. Moreover, this information can be complemented with anthropometric dimensions to establish a quantitative index that relates both the anthropometric dimensions and the exercise frequency.

## Technical Validation

### Data synchronization

For data synchronization, the signals acquired with each WyoFlex armband were regulated by the user with the control buttons in the GUI. In this particular case, the recording of the data is synchronized with the video tutorial. That is, the data recording starts once the video tutorial’s introduction section is finished. Regarding the sampling frequency for the acquisition of the sEMG signals, the authors performed quantification of the latency communication stages of the adopted WyoFlex device with a FireBeetle ESP32 microcontroller implementing the UDP communication protocol.

### Example with a classification algorithm

To provide an example applying the proposed dataset, we implemented a conventional classification algorithm based on artificial neural networks (ANNs) through the Neural Net Pattern Recognition toolbox in Matlab^®^, which has a two-layer feedforward network with hidden sigmoid neurons and softmax output neurons. In this case, the six classes for the classification algorithm have been proposed. The selected classes were M1 (“Flexion”), M2 (“Extension”), M3 (“Ulnar deviation”), M4 (“Radial deviation”), M6 (“Power grip”), and M8 (“Precision grip”).

Here, it should be emphasized that the proposed example considers a subset of the complete dataset since the classification algorithm considers 15 participants who executed three cycles of six movements with each of their forearms. Therefore, the example comprises 540 input signals to the ANNs (90 signals per movement). In order to evaluate the proposed subset of the dataset without the need to compute numerous principal characteristics. Then, before implementing the classification algorithm, this work suggests a pre-processing stage, which consists of computing vectors containing the average (each 10 Hz) of the Fast Fourier Transform (FFT) of each signal obtained by using the Matlab^®^ function fft().

**Fig. 9:**
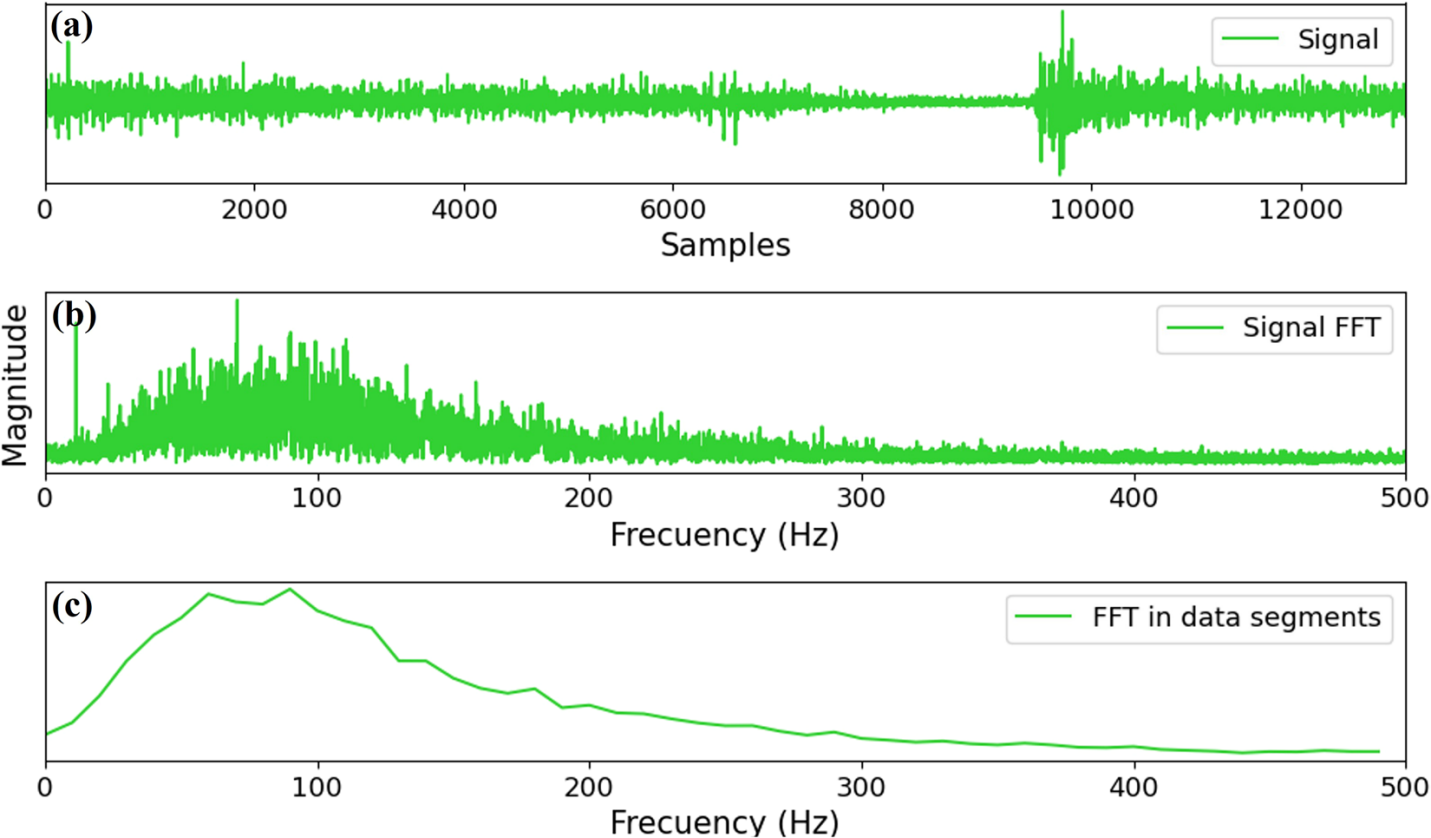
Example of the signal obtained from the file P3C1S1M1F1O2 with its corresponding FFT and FFT average.

In order to complement the pre-processing stage description, Fig. 9a shows the sEMG signal obtained from the file P3C1S1M1F1O2 of the dataset, which corresponds to the patient number three (P3), cycle one (C1), sensor one (S1), flexion movement (M1), right forearm (F1), without offset in the signal (O2). Then, the FFT of the signal was obtained, as can be observed in Fig. 9b. Here, the first five elements of the vector describing the FFT of the signal were omitted to avoid low-frequency components (see^29^ for most information). Therefore, the obtained FFT is a vector with 6495 elements (after skipping the first five elements) distributed in a frequency range from 0 to 500 Hz (see Fig. 9b). Notice that for most processing systems, it could be difficult to process a set of vectors with that amount of elements. Therefore, to avoid the need to compute vectors with an excessive number of elements, the average of the FFT in data segments at each 10 Hz was obtained as a vector with only 50 elements (see Fig. 9c). Notice that all the described pre-processing stage is provided to the user in a Matlab^®^ script that is explained in detail in the subsequent paragraphs.

**Fig. 10:**
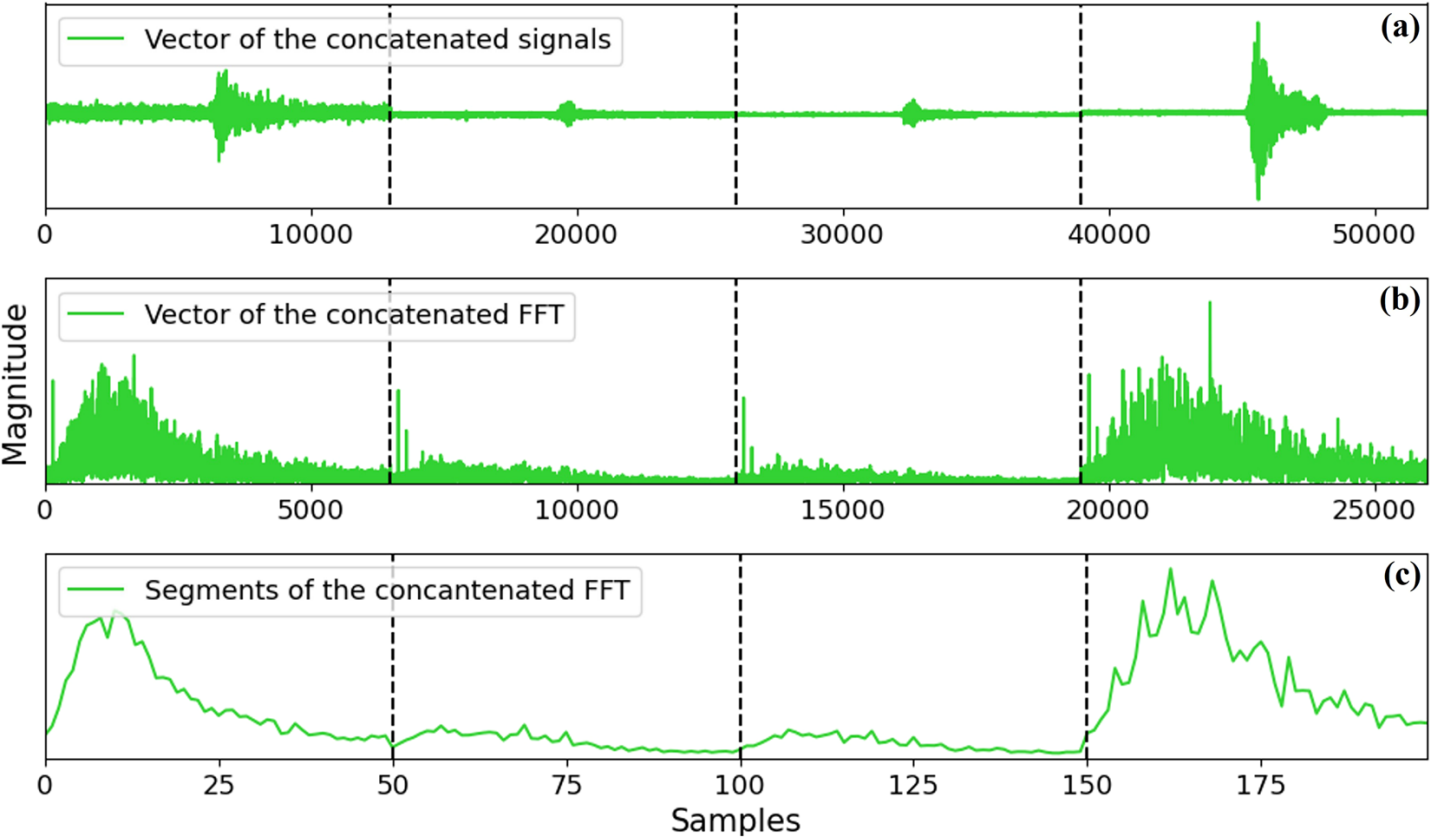
Example of the concatenated signals of the four sensors with its corresponding concatenated FFT and FFT average.

Once the pre-processing stage is described, the input for the ANN implementation should be generated. Then, the Matlab^®^ script (*Example_Classification.m* available in *figshare*^28^) must be implemented to obtain the input Matrix of the classification algorithm. Here, the script generates a vector containing the signals of the four sensors (S1, S2, S3, and S4) per each movement in a concatenated form as shown in Fig. 10a. After that, a vector with the concatenated FFT of each signal is generated as shown in Fig. 10b. Finally, the Matlab^®^ script is in charge of create a vector with the average of the FFT (each 10 Hz) as can be seen in Fig. 10c. As a result of the *Example_Classification.m* implementation, the user gets a matrix with dimensions of 540×200 where the value 540 denotes the concatenated FFT average of the signals, whereas the value of 200 is the number of elements in concatenated FFT average signal. Then, the matrix input was evaluated with different numbers of neurons (34 and 49).

**Fig. 11:**
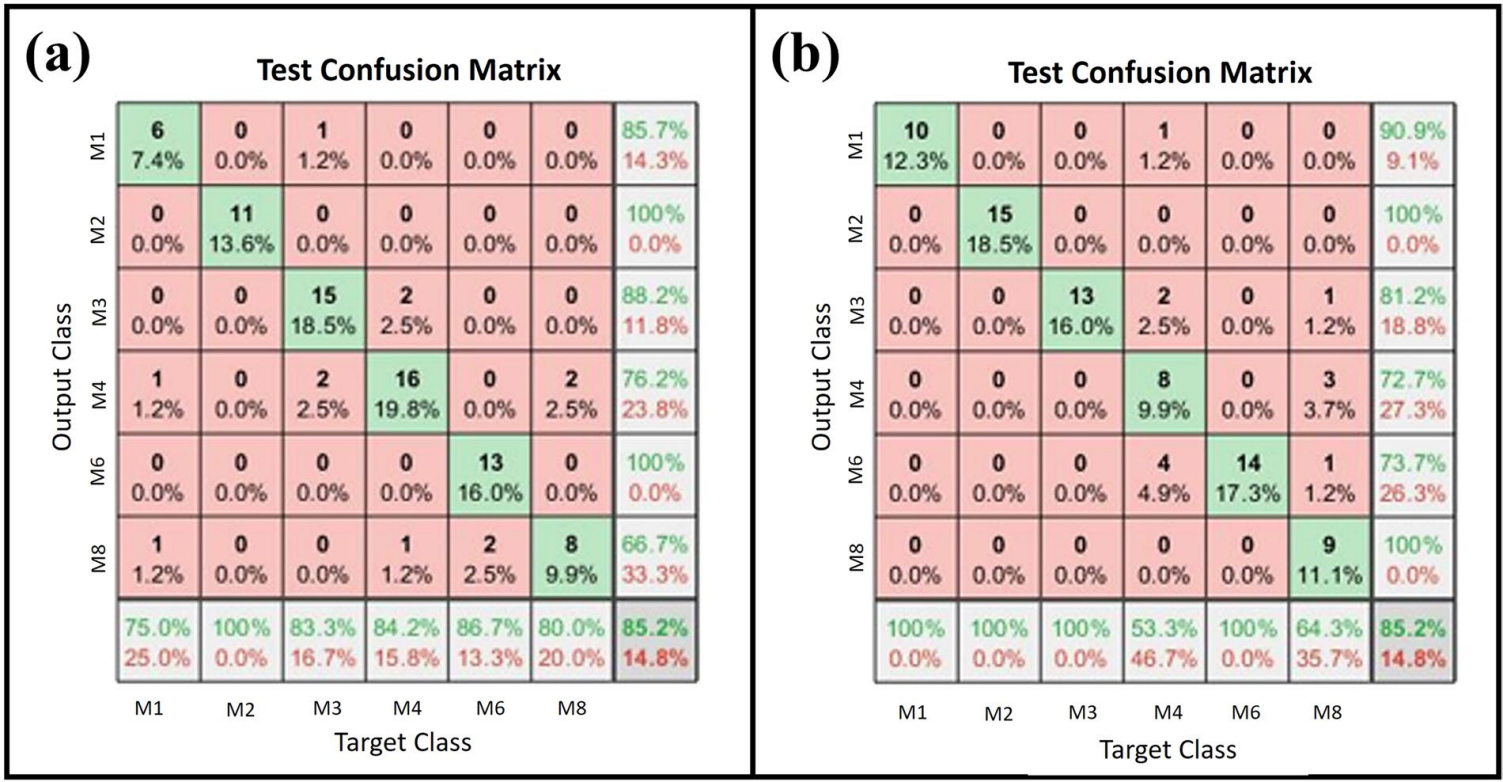
Test confusion matrices (**a**) Confusion matrix for 34 neurons, (**b**) Confusion matrix for 49 neurons.

The results of the classification algorithm are depicted in Fig. 11. From this figure, it can be corroborated that a classification percentage of 85.2% was obtained for both cases (34 and 49 neurons, respectively). Here, it should be emphasized that the proposed toolbox is a standard tool for classification. Then, it could not be the best option regarding classification algorithms for sEMG signals. The previous justifies the obtained percentages. Nevertheless, the dataset can be used to test new classification algorithms.

To summarize all the described in this example, a video tutorial (*Example_Classification_Tutorial.mp4* available in *figshare*^28^) is provided. This video offers a step-by-step explanation to implement the classification through the Neural Net Pattern Recognition toolbox in Matlab^®^.

### Example based on a non-parametric identification by differential neural networks

This subsection provides an example of a nonlinear identification algorithm to test the dataset. This algorithm is based on the so-called differential neural networks (DNNs). The main objective of this technique is to represent a complex nonlinear system by a set of nonlinear ordinary differential equations, that is, to obtain a suitable non-parametric mathematical model. In this particular case, the states of the identification algorithm are the sEMG signals obtained from the sensors *S*1, *S*2, *S*3, and *S*4 for one of the movements in the dataset. Then, the objective is to provide a mathematical model which can reproduce the dynamics in the muscle to generate new sEMG signals. Here, the readers are referred to the book entitled *“Differential neural networks for robust nonlinear control: identification, state estimation, and trajectory tracking”*^30^ for more details about the technique.

Let us consider that a general mathematical form describing the set of the sEMG signals is given by,

2 $$\mathop{x}\limits^{{}^{\cdot }}=f(x,t),\,x(0)={x}_{0},$$

where *x* ∈ ${\mathbb{R}}$^4^ is a vector containing the measured sEMG stimulus stored in the dataset, ${\mathbb{R}}$ is the set of real numbers, *x*_0_ is the initial condition of the differencial equation given in (2), *f*: ${\mathbb{R}}$^4^ × ${\mathbb{R}}$_+_→${\mathbb{R}}$^4^ is the nonlinear function that describes the behavior of the sEMG measurements in time, with ${\mathbb{R}}$_+_ representing the set of positive real numbers. Based on the DNNs theory, it is assumed that the system (2) accepts the following representation

3 $$\mathop{x}\limits^{{}^{\cdot }}=Ax+{W}^{\ast }\sigma (x)+\mathop{f}\limits^{ \sim }(x),$$

with *A* ∈ ${\mathbb{R}}$^4 × 4^ being a stable matrix and W^*^ ∈ ${\mathbb{R}}$^4 × *l*^ describing the weights of the neural network approximation. Here, it is assumed that these parameters approximate the sEMG signal with the best (in some sense) quality, *σ* : ${\mathbb{R}}$^4^→${\mathbb{R}}$^*l*^ denotes the activation functions, which are selected as sigmoid ones, that is *σ* = [*σ*_1_ ⋯ *σ*_*l*_]^T^,

4 $${\sigma }_{i}=\frac{{a}_{i}}{1+{b}_{i}{e}^{{c}_{i}^{{\rm{T}}}x}},i=\left\{1:l\right\},$$

where *a*_*i*_ ∈ ${\mathbb{R}}$, *b*_*i*_ ∈ ${\mathbb{R}}$, and *c*_*i*_ ∈ ${\mathbb{R}}$^4^ denote the parameters of the sigmoidal function.

In Eq. (3), $\widetilde{f}:{{\mathbb{R}}}^{4}\to {{\mathbb{R}}}^{4}$ denotes the modeling error, which comes from the finite number of sigmoid functions implemented to approximate the sEMG stimulus. The objective of this theory is to obtain a suitable identifier that approximate the sEMG stimulus through a non-parametric algorithm represented by a DNN identifier. This identifier is proposed as follows

5 $$\dot{\hat{x}}=A\hat{x}+W\sigma (\hat{x}),$$

notice that it has a similar structure like system (3), $\widehat{x}\in {{\mathbb{R}}}^{4}$ is the estimated value of *x*, *A* and *σ* have the same meaning as in Eq. (3), *W* are the estimated weights that obey the following learning law

6 $$\mathop{W}\limits^{{}^{\cdot }}=\,-\,kP\Delta \sigma {(\hat{x})}^{{\rm{\top }}}.$$

Here, $\Delta =\widehat{x}-x$ is named as the identification error, *k* is a positive constant called the learning coefficient, *P* = *P*^T^ > 0 is a positive definite and symmetric matrix^30^. The application of the so-called Lyapunov theory yields in the derivation of the learning laws described in (6).

In order to implement the example, a Matlab^®^ script (*Example _Identification.m* available in *figshare*^28^) must be executed. Then, before implementing the script, the user should take into account that the example works only with the signals in the VOLTAGE DATA folder of the dataset. Therefore, it is not suitable to implement the code with the signals in the DIGITAL DATA folder. Also, it should be emphasized that the performance obtained with the provided algorithm depends on adjusting some parameters. Here, the user is in charge of tuning in the code, by trial and error, the variables *P* and *k*.

Once the user runs the code, the algorithm’s instructions are displayed. The next step is selecting the folder where the sEMG dataset is stored on their computer. Then, the user must provide as inputs for the script the following parameters, participant number, number of cycles, movement number, arm side, and if the signal considers offset or not. As the output of the algorithm, four graphics are obtained. Each of them corresponds to each of the four sensors. In the mentioned figures, two signals are shown (one in color blue and one in color green), where the solid blue line represents the sEMG of the database, whereas the solid green line denotes the estimation obtained with the algorithm for the corresponding sensor.

The parameters used by default in the Matlab^®^ script are

7 $$k=50,\quad A=\left[\begin{array}{cccc}-2000 & 0 & 0 & 0\\ 0 & -1000 & 0 & 0\\ 0 & 0 & -800 & 0\\ 0 & 0 & 0 & -1800\end{array}\right],$$

*σ* is defined as $\sigma ={[{\sigma }_{1}(3,1.2,-2.2{I}_{v}),{\sigma }_{2}(7.5,2.5,-5.5{I}_{v}),{\sigma }_{3}(9,0.6,-6.6{I}_{v}),{\sigma }_{4}(10.5,0.9,-7.7{I}_{v})]}^{{\rm{\top }}}$, with *I*_*v*_ = [1, 1, 1, 1]^T^. The representation *σ*_*i*_(*a*_*i*_, *b*_*i*_, *c*_*i*_) obeys the definition in (4). The initial conditions for the identifier are chosen as $\hat{x}(0)={[0,0.1,0.5,-0.1]}^{{\rm{\top }}}$.

**Fig. 12:**
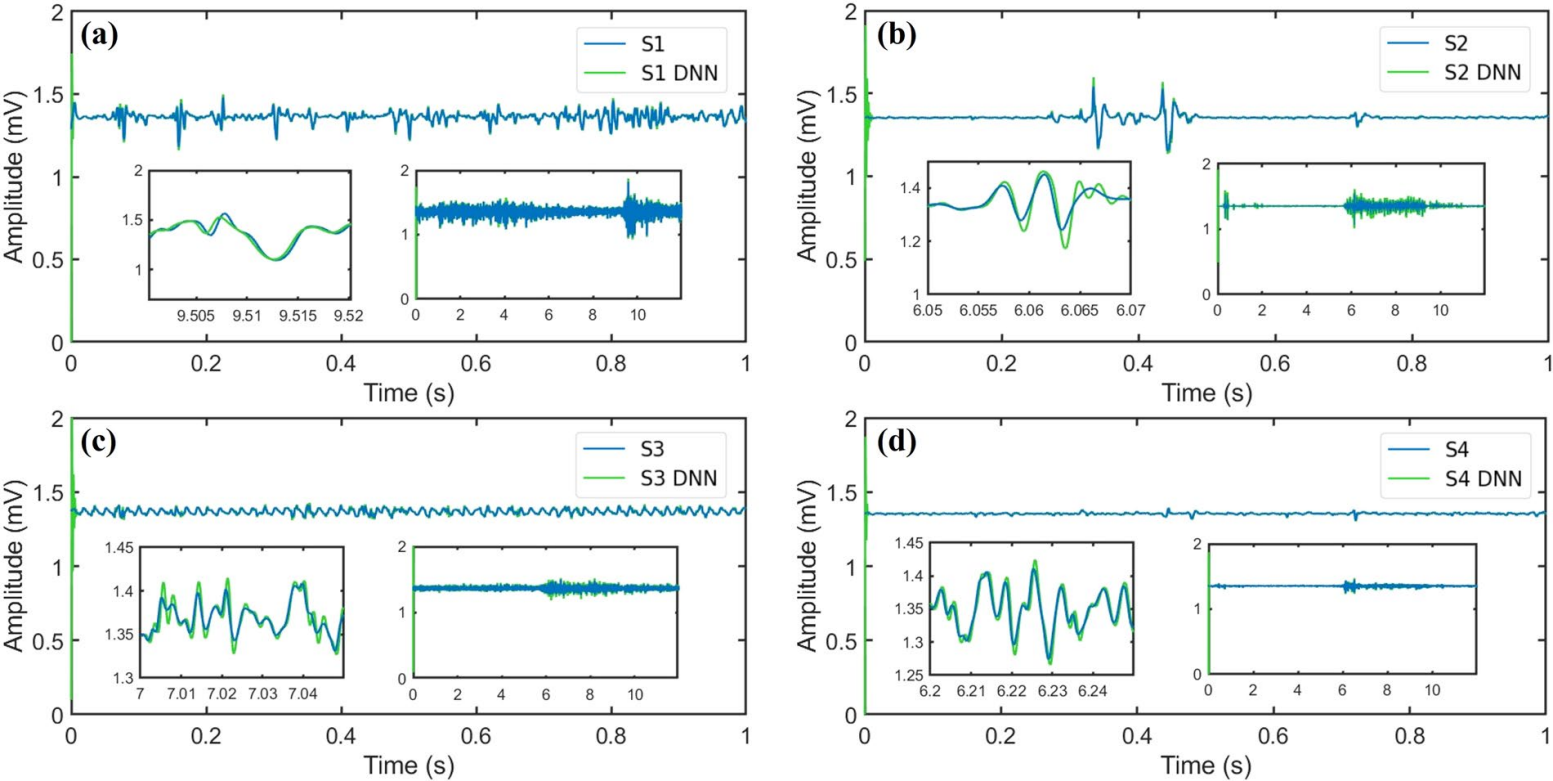
State identification of the states obtained with the WyoFlex device and stored in the dataset. (**a**) Sensor 1, (**b**) Sensor 2, (**c**) Sensor 3, (**d**) Sensor 4.

**Fig. 13:**
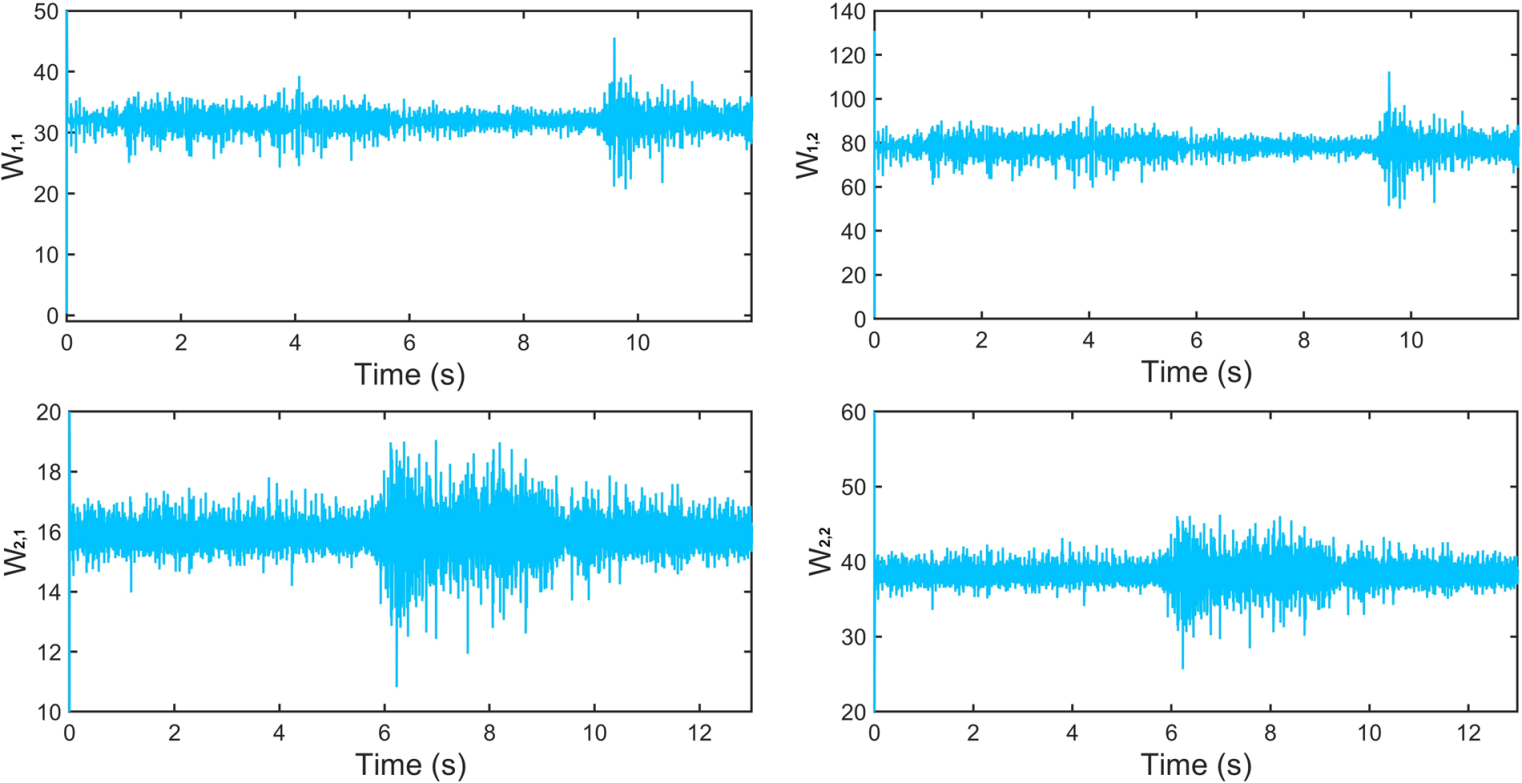
Weights dynamics.

**Fig. 14:**
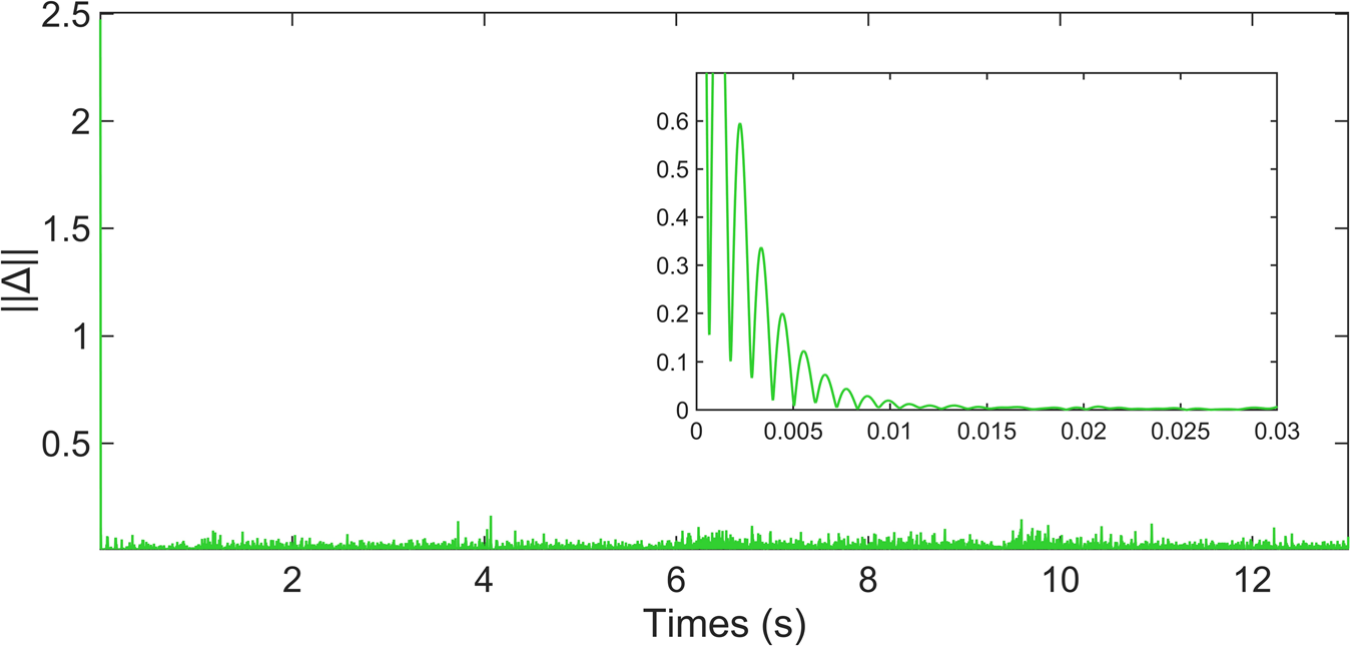
Euclidean norm of the identification error.

The obtained results are shown in Fig. 12, where a), b), c) and d) correspond to the sensors *S*1, *S*2, *S*3 and *S*4, respectively. The blue line represents the real stimulus and the red continuous line is the identification provided by the DNN. Even though the initial conditions were randomly chosen and the high degree of non-linearities, the DNN algorithm identifies the unknown states in less than 0.01 seconds. Figure 13 shows the adaptation of the weights in the neural network. The weights copy the high non-linearities of the sEMG signal. The results showed in Figs. 12, 13 are made in one test of the dataset. However, to visualize the performance of the identifier over the dataset, the DNN identifies all the signals of one movement. To represent the performance of the DNN over the entire database, Fig. 14 shows the Euclidean norm of the identification error. Notice that, the error remains in a small zone around zero.

The applications of DNNs to identify nonlinear systems include the derivation of nonlinear math models that can be applied in the control of active orthoses or prostheses for rehabilitation. In the work developed by Lozano, A. *et al*.^31^, the input to the DNN is the stimulus and the output $\widehat{x}$ is the current movement should be executed by the orthosis. On the other hand, in Llorente-Vidrio, D. *et al*.^32^, the DNN classifies electroencephalographic (EEG) signals. To this end, the input of the DNN are the EEG stimulus and the output $\widehat{x}$ the desired class.

## Supplementary information

Human Data Submission Checklist

## Data Availability

A Matlab^®^ script (*Code_Availability.m* available in *figshare*^28^) and a Python script (*Code_Availability.phy* available in *figshare*^28^) are provided to demonstrate how the dataset can be accessed and how to visualize an sEMG signal. The provided code has a menu where the user can select a specific signal to be visualized. Another code (*Example_Classification.m* available in *figshare*^28^) is provided to access the dataset and obtain the FFT of the dataset. This code can be used to generate an input file to implement a classification algorithm using the Neural Net Pattern Recognition toolbox in Matlab^®^, as explained in the first example of the Technical Validation Section. Also, the code (*Example_Identification.m* available in *figshare*^28^) is provided, this code help to the user to implement an identification algorithm with the signals in the dataset.
